# CRISPR decodes the RNA regulatory network in prostate cancer: A review from mechanisms to precision therapeutics

**DOI:** 10.1016/j.ncrna.2026.05.001

**Published:** 2026-05-28

**Authors:** Song Zhu, Gao Ni, Pinjie Zhang, Yili Yang, Hongxiang He, Junfeng Jiang, Zhang Li

**Affiliations:** a School of Gongli Hospital Medical Technology, University of Shanghai for Science and Technology, Shanghai 200093, China; b Department of Histology and Embryology, College of Basic Medicine, Naval Medical University, Shanghai 200433, China; c Department of Pathogen Biology, Naval Medical University, Shanghai 200433, China; d Department of Pathology, Faculty of Medical Imaging, Naval Medical University, Shanghai, 200433, China

**Keywords:** CRISPR technology, Prostate cancer, RNA regulatory network, Non-coding RNA, Precision medicine

## Abstract

Prostate cancer (PCa) remains one of the most prevalent and lethal malignancies in men worldwide. The pronounced heterogeneity and therapeutic resistance of PCa underscore the critical need to decipher its RNA regulatory network for advancing precision medicine. This review systematically outlines the cutting-edge applications of CRISPR technology in studying the RNA regulatory network of PCa. We highlight the CRISPR systems as a powerful tool for functional decoding, target screening, ultrasensitive diagnostics, and precision intervention, emphasizing their pivotal role in elucidating the functional mechanisms of mRNA, miRNA, circRNA, lncRNA, eRNA, and m^6^A modification. Furthermore, this review synthesizes how these insights not only reveal the core functions of diverse RNA molecules in PCa initiation, progression, drug resistance, and metastasis but also propel the innovation of novel therapeutic strategies, including mRNA vaccines, Cas13-mediated RNA editing, and targeted delivery systems. Finally, we discuss future directions and challenges in developing personalized CRISPR-RNA therapeutics integrated with multi-omics technologies, aiming to provide a theoretical foundation and technical framework for precision medicine in PCa.

**Table:** Abbreviations:

PCa	Prostate cancer
ADT	Androgen deprivation therapy
CRPC	Castration-resistant prostate cancer
CRISPR	Clustered Regularly Interspaced Short Palindromic Repeats
CRISPRi	CRISPR interference
CRISPRa	CRISPR/Cas9 activation systems
SF	Splicing factor
AR	Androgen receptor
AR-V7	Androgen receptor splice variant 7
SF3B2	Splicing factor 3b subunit 2
CE3	Cryptic exon 3
MFAP1	Microfibril Associated Protein 1
POLR2A	RNA Polymerase II Subunit A
LSM3	LSM3 homolog, U6 small nuclear RNA associated
DHX16	DEAH-box helicase 16
HNRNPL	Heterogeneous nuclear ribonucleoprotein L
DNMT3a	DNA methyltransferase 3 alpha
eIF4A1	Eukaryotic translation initiation factor 4A1
BRD2	Bromodomain containing 2
YAP1	Yes-associated protein 1
CTGF	Connective tissue growth factor
ARID5A	AT-rich interaction domain 5 A
IL-6	Interleukin 6
H3K4me3	Trimethylation of lysine 4 on histone H3
INTS14	Integrator complex subunit 14
sLZIP	small leucine zipper protein
GR	Glucocorticoid receptor
MMP-13	Matrix metallopeptidase 13
ERβ	Estrogen receptor beta
DACH1	Dachshund family transcription factor 1
PURα	Purine-rich element binding protein alpha
RBX1	Ring-box protein 1
CHD1	Chromodomain Helicase DNA Binding Protein 1
PRMT7	Protein arginine methyltransferase 7
FoxK1	Forkhead box K1
POLRMT	Mitochondrial RNA polymerase
STK4	Serine/threonine kinase 4
PP2A	Protein phosphatase 2 A
CYR61	Cysteine-rich angiogenic inducer 61
PHF8	PHD finger protein 8
HIF1A	Hypoxia-inducible factor 1 alpha
KDM3A	Lysine demethylase 3 A
TRPM4	Transient receptor potential cation channel subfamily M member 4
4-MI	4-methylindole
KLK3	Kallikrein-related peptidase 3
HVEM	Herpesvirus entry mediator
BTLA	B and T lymphocyte associated
PD-1	Programmed cell death protein 1
TRAF6	TNF receptor associated factor 6
Wnt3a	Wnt family member 3a
MPRA	Massively parallel reporter assay
IGF1R	Insulin-like growth factor 1 receptor
ARE	AU-rich element
SNPs	Single nucleotide polymorphisms
MSMB	Microseminoprotein beta
CTCF	CCCTC-binding factor
KCNN3	Potassium calcium-activated channel subfamily N member 3
KRT78	Keratin 78
LNPs	Lipid nanoparticles
E2F8	E2F transcription factor 8
HOXB13	Homeobox protein B13
QDT	Qingdai Tang
NOS3	Nitric oxide synthase 3
TGFB1	Transforming growth factor beta 1
NCOA2	Nuclear receptor coactivator 2
PI3K	Phosphoinositide 3-kinase
AKT	Protein kinase B
PD-L1	Programmed cell death ligand 1
PTEN	Phosphatase and tensin homolog
MiRNAs	MicroRNAs
TMEM97	Transmembrane protein 97
MAPK	Mitogen-activated protein kinase
ERK	Extracellular signal-regulated kinase
EMT	Epithelial-mesenchymal transition
t-NEPC	Treatment-induced neuroendocrine prostate cancer
SOX2	SRY-box transcription factor 2
HMGA2	High mobility group AT-hook 2
AIFM2	Apoptosis inducing factor mitochondria associated 2
RECK	Reversion-inducing cysteine-rich protein with Kazal motifs
PDCD4	Programmed cell death 4
MMP9	Matrix metallopeptidase 9
EGLN3	egl-9 family hypoxia-inducible factor 3
BCLAF1	BCL2 associated transcription factor 1
PUMA	p53 upregulated modulator of apoptosis
BAK1	BCL2 antagonist/killer 1
CDK13	Cyclin-dependent kinase 13
ATM	Ataxia telangiectasia mutated
ULK2	UNC-51 like autophagy activating kinase 2
mTOR	Mechanistic target of rapamycin
TP53INP1	Tumor protein p53 inducible nuclear protein 1
RAB27A	RAS-associated protein RAB27A
HRCA	Hyperbranched Rolling Circle Amplification
POCT	Potential for point-of-care testing
Cx-43	Connexin 43
PTPN4	Protein tyrosine phosphatase non-receptor type 4
CAR-T	Chimeric antigen receptor T-cell
CircRNAs	Circular RNAs
ceRNAs	Competitive endogenous RNAs
ZNF609	zinc finger protein 609
CCNB2	Cyclin B2
IGF2BP3	Insulin-like growth factor 2 mRNA binding protein 3
HDAC4	Histone deacetylase 4
FMN2	Formin 2
huR	Human antigen R
KLF2	Krüppel-like factor 2
RMST	Rhabdomyosarcoma 2 associated transcript
MID1	Midline 1
KDM4A	Lysine demethylase 4 A
AURKA	Aurora kinase A
LncRNAs	Long non-coding RNAs
TTTY15	Testis-specific transcript Y 15
CDK6	Cyclin-dependent kinase 6
FN1	Fibronectin 1
MALAT1	Metastasis associated lung adenocarcinoma transcript 1
eQTLs	Expression quantitative trait loci
PCAT19	Prostate cancer associated transcript 19
HOXA2	Homeobox protein A2
SNHG11	Small nucleolar RNA host gene 11
NCOA4	Nuclear receptor coactivator 4
GWAS	Genome-wide association study
HOTTIP	HOXA distal transcript
PCAT1	Prostate cancer associated transcript 1
PCA3	Prostate cancer antigen 3
DRD2	Dopamine receptor D2
AMPK	AMP-activated protein kinase
eRNAs	Enhancer RNAs
TADs	Topologically associating domains
LTF	Lactoferrin
DNMT1	DNA methyltransferase 1
PSA	Prostate-specific antigen
P-TEFb	Positive transcription elongation factor b
hnRNPU	Heterogeneous nuclear ribonucleoprotein U
EGR1	Early growth response protein 1
CCND1	Cyclin D1
SNAPC1	Small nuclear RNA activating complex polypeptide 1
eRNA-QTLs	eRNA quantitative trait loci
m6A	N^6^-methyladenosine
YTHDF1	YTH N^6^-methyladenosine RNA binding protein 1
PLK1	Polo-like kinase 1
YTHDF2	YTH N^6^-methyladenosine RNA binding protein 2
LHPP	Phospholysine phosphohistidine inorganic pyrophosphate phosphatase
NKX3-1	NK3 homeobox protein 1
METTL3	Methyltransferase-like protein 3
HNRNPA2B1	Heterogeneous nuclear ribonucleoprotein A2/B1
FTO	Fat mass and obesity-associated protein
ALKBH5	AlkB homolog 5

## 1 Introduction

Prostate cancer (PCa) ranks among the most prevalent malignancies in men worldwide. Although relatively effective clinical treatments, such as surgery, radiotherapy, and androgen deprivation therapy (ADT) are available, many patients still eventually progress to castration-resistant prostate cancer (CRPC). The high heterogeneity and therapy resistance of the tumor lead to a significant worsening of patient prognosis [1,2]. Studies have shown that this heterogeneity is closely linked to the intricate RNA regulatory network within cells. In this network, RNA not only serves as a messenger of genetic information but also acts as a key regulator of gene expression [3]. Specifically, molecules such as mRNA, miRNA, circRNA, lncRNA, and eRNA form a multi-layered regulatory system. In prostate cancer, aberrant mRNA splicing and translational reprogramming directly drive tumor progression. miRNAs function as post-transcriptional hubs, finely modulating oncogene and tumor suppressor networks. circRNAs and lncRNAs participate in tumorigenesis and development through mechanisms including molecular sponging and chromatin regulation. eRNA contributes to tumor progression by mediating chromatin interactions and dynamically regulating oncogenic transcriptional programs [[4], [5], [6], [7]]. In addition, RNA molecules carry various reversible chemical modifications, among which m^6^A plays a central role in gene expression regulation by dynamically controlling RNA stability, splicing, translation, and degradation [8]. Together, these RNA molecules form a multi-layered and tightly controlled regulatory system. Dysregulation of this network is a key driver of prostate cancer evolution. However, its complexity involves intricate molecular interactions, context dependence, and spatiotemporal dynamics, which makes it difficult to systematically resolve using conventional techniques.

In recent years, advances in clustered regularly interspaced short palindromic Repeats (CRISPR) gene-editing technology have provided new perspectives for deciphering this network. CRISPR technology has evolved into a versatile platform beyond DNA editing. It now encompasses several types of tools, including DNA-targeting editors for precise genome modification, functional genomics screeners for high-throughput gene discovery, nucleic acid detectors for ultra-sensitive diagnostics, and RNA-targeting perturbators for manipulating transcripts. This continuously expanding CRISPR toolkit enables researchers to analyze the functions and investigate the mechanisms of specific RNA molecules with unprecedented precision and throughput in a genome-wide context [[9], [10], [11], [12]].

**Fig. 1:**
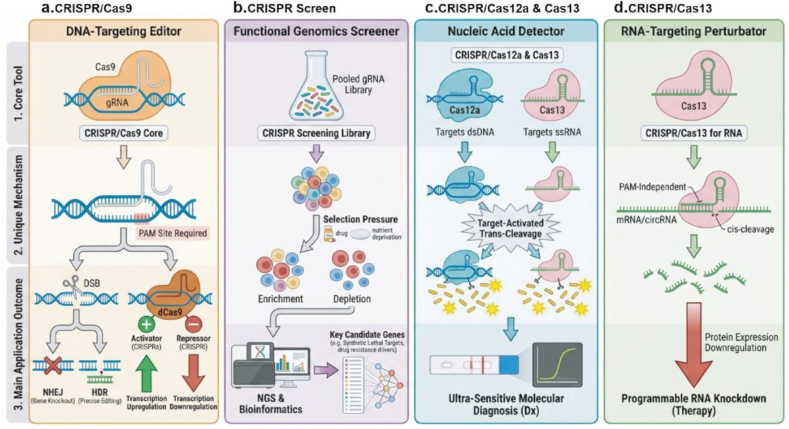
**The Multifunctional CRISPR Toolkit in Modern Biology.** (a) Exemplified by Cas9, this module enables precise knockout, insertion, or replacement of genomic sequences for creating disease models and gene therapy. (b) Utilizing genome-wide CRISPR knockout/activation libraries, this module allows for high-throughput screening of genes essential for specific phenotypes. (c) Based on the “collateral cleavage” activity of Cas12/Cas13, this module achieves ultra-sensitive detection of specific DNA/RNA sequences for molecular diagnostics. (d) Employing effectors like Cas13 or dCas13, this module enables knockdown, localization, or modification of RNA molecules without altering the genome, facilitating functional studies and targeted therapies.

CRISPR technology originates from bacterial immune systems. The system consists of two core components [13], a Cas nuclease and a guide RNA. The guide RNA uses sequence complementarity to direct the Cas protein to precisely target specific nucleic acid sequences [9], enabling specific recognition and cleavage. With technological advances, CRISPR has evolved into a versatile platform with diverse functions (Fig. 1). CRISPR interference (CRISPRi) uses an inactivated Cas protein fused with transcriptional repressors to interfere with and downregulate gene expression [14]. CRISPR activation (CRISPRa) employs an inactivated Cas protein fused with transcriptional activators to target and activate specific genes [15]. CRISPR screen uses genome-scale guide RNA libraries for high-throughput screening, enabling systematic identification of functional genes associated with specific phenotypes [16]. In addition, different Cas enzymes possess distinct functional characteristics. Cas9 primarily acts on DNA and achieves genome editing through double-strand breaks [17]. Cas12 has both DNA cleavage and collateral cleavage activities [18], making it suitable for nucleic acid detection. Cas13 targets RNA molecules and can be used for transcript knockdown, editing, and detection [19].

**Fig. 2:**
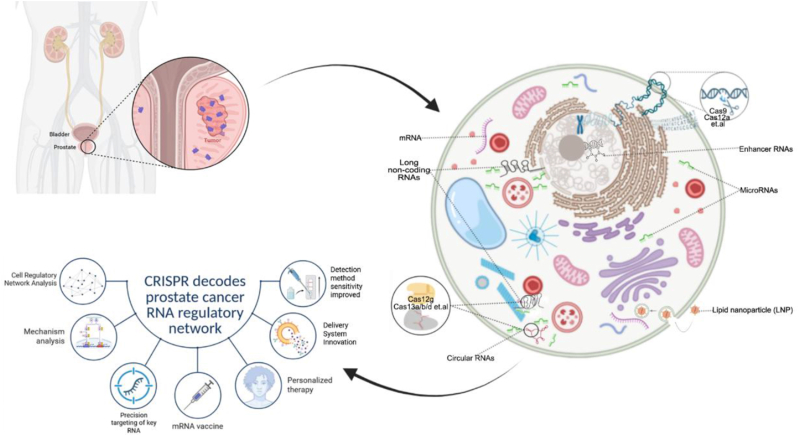
**CRISPRDecodesProstateCancer RNARegulatoryNetwork.** CRISPR effector proteins are guided to specific regulatory nodes. They target DNA or RNA molecules to reveal their functional roles in prostate cancer progression. This approach maps interactions between RNA molecules. It provides a systematic perspective for identifying key oncogenic or tumor-suppressive pathways. For example, Cas9 or Cas12a enables precise DNA cleavage for gene function studies. Cas13a, Cas13b, Cas13d, or Cas12g targets RNA directly for transcript degradation or RNA-based diagnosis.

This review focuses on the use of CRISPR technology to study RNA-level regulatory networks, with an emphasis on mRNA, miRNA, circRNA, lncRNA, eRNA, and m^6^A modification (Fig. 2). It systematically decodes their critical roles in prostate cancer initiation, progression, and treatment. It covers how CRISPR tools interrogate mRNA splicing, miRNA circuits, non-coding RNA functions, eRNA-mediated regulation, and m^6^A modifications. These discussions highlight key advances and future directions.

**Fig. 3:**
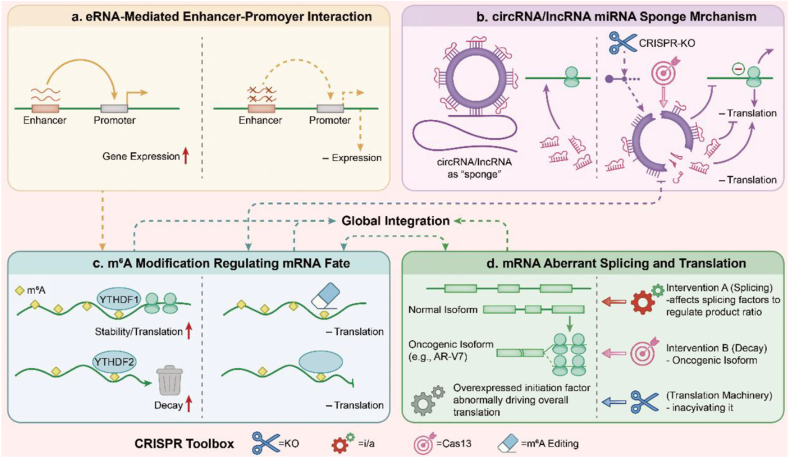
**RNA Regulatory Mechanisms and Their Targeted Perturbation by CRISPR/Cas Systems.** (a) eRNAs facilitate contact between enhancers and promoters, enhancing gene expression. CRISPR perturbation disrupts these interactions, reducing transcription. (b) CircRNAs/lncRNAs sequester miRNAs, preventing them from binding targets. CRISPR-KO releases miRNAs, restoring their regulatory function and affecting translation. (c) m^6^A marks recruit YTHDF proteins, enhancing mRNA stability and translation. Removal or modification of m^6^A sites by CRISPR tools alters mRNA fate, increasing decay. (d) Variants affect splicing accuracy, producing oncogenic isoforms. CRISPR-based interventions adjust splicing factors or inhibit RNA machinery, rebalancing translation.

To decode these diverse RNA species, CRISPR-based approaches have established several complementary strategies of RNA-level intervention. As summarized in Fig. 3, these include disrupting enhancer–promoter interactions by targeting eRNAs, releasing sequestered miRNAs through circRNA or lncRNA knockout, altering transcript stability via site-specific m^6^A editing, and correcting oncogenic mRNA splicing. These mechanistic paradigms run through the entire review, providing an integrated framework for the following sections, which examine in detail how CRISPR technology has been applied to each class of RNA molecules in prostate cancer research, and briefly point to the translational directions that emerge from these insights (Fig. 3).

## 2 CRISPR-mediated mRNA regulation and therapy

As the central carrier of genetic information, mRNA's aberrant expression, splicing, and translation are key factors [20,21] driving PCa progression. This section will explore how CRISPR technology is being used to investigate the complex regulatory network of mRNA in PCa and to facilitate its translation into clinical therapies.

### 2.1 CRISPR reveals mRNA splicing variants and translation

Splicing factor (SF)-mediated alternative mRNA splicing is a key mechanism underlying therapy resistance in PCa. A representative example is the androgen receptor (AR) [22] splice variant 7 (AR-V7). Due to aberrant mRNA splicing, AR-V7 lacks the ligand-binding domain, enabling it to constitutively activate downstream genes independent of androgen, which constitutes a core mechanism of resistance to androgen deprivation therapy in PCa [23,24]. Studies have revealed that CRISPR/Cas9, coupled with a GFP reporter system, identified that the splicing factor 3b subunit 2 (SF3B2) [25] binds specific motifs on AR pre-mRNA, promoting the inclusion of the cryptic exon 3 (CE3) and thereby driving the generation of the AR-V7 splice variant. Research by Whitton B. et al. [26] employed CRISPR knockout to demonstrate a compensatory balance between V-ATPase subunits ATP6V1C1 and V1C2 in regulating the activity of AR variants. Walker et al. [27] utilized a customized CRISPR library targeting splicing factors in a screen and found that knockout of microfibril associated protein 1 (MFAP1) significantly reduced AR-V7 mRNA levels. This intervention synergized with RNA polymerase II subunit A (POLR2A) inhibitors to kill tumor cells and enhanced radiosensitivity. Furthermore, database screening based on CRISPR-Score identified novel splicing factors such as LSM3 homolog, U6 small nuclear RNA associated (LSM3) and DEAH-box helicase 16 (DHX16) as essential genes for the cell cycle. These factors [28] promote PCa progression by modulating Cyclin protein expression to drive cell cycle progression. Genome-wide CRISPR/Cas9 screening revealed that the RNA-binding protein heterogeneous nuclear ribonucleoprotein L (HNRNPL) is a key factor essential for prostate cancer growth. HNRNPL [29] primarily binds CA-repeat sequences within pre-mRNA introns and 3′UTRs, regulating AR alternative splicing and thereby promoting tumor progression.

Studies indicate that aberrant expression of translation initiation factors can drive PCa progression through non-canonical pathways, specifically via epigenetic regulation. Wang et al. [30] designed dCas9- DNA methyltransferase 3 alpha (DNMT3a) to target and methylate the eukaryotic translation initiation factor 4A1 (eIF4A1) promoter, using CRISPR/Cas9 technology to confirm that eIF4A1 drives PCa progression at the translational level through the bromodomain containing 2 (BRD2)-MYC axis. In summary, therapy resistance and progression in PCa are regulated by mechanisms including mRNA splicing variants and translational reprogramming, and the elucidation of this complex network relies indispensably on CRISPR technology.

### 2.2 CRISPR identifies key transcriptional networks

In the functional analysis of key transcription factors, CRISPR/Cas9 and CRISPRa [31] have become powerful tools for directly validating the mechanisms of specific transcription factors in PCa progression. Kim et al. [32] used CRISPR/Cas9 technology to directly verify that yes-associated protein 1 (YAP1) drives transcription by binding to the CTGF promoter, and its activity can be inhibited by the drug GV1001. The Ikeuchi team employed CRISPR technology to knockout and activate AT-rich interaction domain 5 A (ARID5A), revealing that it activates interleukin 6 (IL-6) [33] transcription by recruiting RNA polymerase II and maintaining trimethylation of lysine 4 on histone H3 (H3K4me3) histone modification. Furthermore, Tanaka Y. et al. [34] utilized a CRISPRa library screen and identified integrator complex subunit 14 (INTS14) as a novel positive regulator of MYC transcription. CRISPR/Cas9 technology further confirmed that small leucine zipper protein (sLZIP) [35] drives CRPC metastasis and antagonizes glucocorticoid receptor (GR) function by activating matrix metallopeptidase 13 (MMP-13) transcription. Estrogen receptor beta (ERβ) loss leads to excessive activation of AR signaling through downregulation of dachshund family transcription factor 1/purine-rich element binding protein alpha (DACH1/PURα) [36], providing a theoretical basis for ERβ agonists to reverse precancerous lesions.

CRISPR genome-wide screening technology provides a global view of the core genes governing PCa transcriptional programs. Using this technology, ring-box protein 1 (RBX1) was identified as a top dependency gene in 17p-deficient CRPC, maintaining POLR2A [37] ubiquitination and short-lived mRNA synthesis. Chromodomain helicase DNA binding protein 1 (CHD1) [38,39] loss triggers transcriptional reprogramming leading to enzalutamide resistance. High-throughput CRISPR screening based on the GeCKO v2 library confirmed that protein arginine methyltransferase 7 (PRMT7), by methylating the transcriptional cofactor forkhead box K1 (FoxK1) [40] and regulating downstream adhesion molecule expression, thereby inhibiting cell metastasis, is a key target for mCRPC invasion.

CRISPR technology has also revealed various non-canonical transcriptional regulatory mechanisms. CRISPR knockout of mitochondrial RNA polymerase (POLRMT) led to decreased mitochondrial transcripts and induced Caspase-9-dependent apoptosis, a phenotype that can be mimicked by the small molecule inhibitor IMT1 [41]. CRISPR technology combined with siRNA elucidated that AR signaling promotes YAP1 dephosphorylation and nuclear translocation through the serine/threonine kinase 4/protein phosphatase 2 A (STK4/PP2A) axis [42], thereby activating target genes such as cysteine-rich angiogenic inducer 61 (CYR61)/CTGF. CRISPR knockout of the histone demethylase PHD finger protein 8 (PHF8) disrupts hypoxia signaling response by reducing the transcription levels of hypoxia-inducible factor 1 alpha (HIF1A) and lysine demethylase 3 A (KDM3A) [43]. CRISPR/Cas9 technology confirmed that p53 loss promotes cell cycle progression by upregulating transient receptor potential cation channel subfamily M member 4 (TRPM4) [44]. 4-methylindole (4MI) can directly inhibit AR transcription of kallikrein-related peptidase 3 (KLK3) [45].

CRISPR technology has also extended the analysis of transcriptional regulatory networks to immune checkpoints. Using CRISPR/Cas9 technology to knockout the immune checkpoint molecule herpesvirus entry mediator (HVEM), NanoString immune gene profiling revealed downregulated mRNA expression of the T-cell co-inhibitory receptor B and T lymphocyte associated (BTLA) [46], enhancing the efficacy of anti-programmed cell death protein 1 (PD-1) therapy. CRISPR/Cas9 combined with siRNA confirmed that TNF receptor associated factor 6–Wnt family member 3a (TRAF6–Wnt3a) [47] drives cancer cell invasion through LRP5/β-catenin transcription. These findings demonstrate that CRISPR technology plays a crucial role in systematically identifying key molecules within the transcriptional regulatory hierarchy.

### 2.3 CRISPR validates functional mutations in non-coding RNAs

Studies indicate that the 3′UTR influences mRNA degradation and translation efficiency, and its mutations may be associated with prostate cancer progression [48]. Schuster et al. using an approach that combined massively parallel reporter assay (MPRA) technology with CRISPR base editing, developed two complementary large-scale parallel reporter assays to screen a vast number of genomes from patients with advanced prostate cancer. They identified genes with high-frequency mutations in the 3′UTR region, such as ZWILCH (chr15:66548998 A→G) and insulin-like growth factor 1 receptor (IGF1R, chr15:98958058 G→A). The study found that the mutation in ZWILCH disrupts an AU-rich element (ARE), thereby relieving NCL/AUF1-mediated translational inhibition and increasing its protein expression by 45% [49]. The mutation in IGF1R [49] stabilizes the RNA stem-loop structure, enhancing translation efficiency. Experiments using CRISPR base editing to introduce patient-derived mutations [49] into PC3 cells confirmed a significant increase in translation efficiency. Further investigation into the biological functions of these regions not only helps to reveal the pathogenesis of prostate cancer but also provides potential important targets for developing new diagnostic and therapeutic strategies.

Single nucleotide polymorphisms (SNPs) refer to variations of a single nucleotide in the genome and are widely present in human populations [50,51]. Wang et al. using a CRISPR/Cas9-mediated dual-gRNA deletion strategy, confirmed that the risk SNPs rs7098889 and rs10993994 influence microseminoprotein beta (MSMB) mRNA [52] expression by regulating enhancer and promoter activity, and are associated with poor prognosis in PCa. Guo et al. utilized CRISPR technology to delete CCCTC-binding factor (CTCF) chromatin loop anchor regions near non-coding SNPs associated with prostate cancer risk, revealing that these SNPs can disrupt CTCF-mediated chromatin looping, thereby relieving transcriptional repression of key genes such as potassium calcium-activated channel subfamily N member 3 (KCNN3) and keratin 78 (KRT78) [53] and promoting prostate carcinogenesis.

### 2.4 CRISPR innovations in mRNA therapy and delivery

mRNA-based therapeutic strategies represent a current research frontier, and CRISPR technology plays a crucial role in advancing their clinical translation by optimizing delivery systems and developing novel editors [54,55]. mRNA vaccines are a novel vaccine technology [56,57]. Personalized neoantigen mRNA vaccines encapsulated in lipid nanoparticles (LNPs), such as BNT112 [58], have shown the potential to induce potent immune responses in clinical trials. DC vaccines, loaded with tumor mRNA via electroporation and used as adjuvant therapy in NCT01197625, achieved a long-term biochemical recurrence-free survival of over 8 years in 55% of postoperative patients. Furthermore, Cao et al. [59] found that the combined use of 5T4 and CD70 mRNA-LNPs can significantly enhance both cellular and humoral immunity, representing a promising immunotherapeutic strategy for prostate cancer.

However, mRNA vaccine strategies face two major challenges. The high cost of neoantigen screening and the lengthy preparation cycle for personalized vaccines [60]. To address the high cost of neoantigen screening for mRNA vaccines, Tabibian et al. proposed using CRISPR technology to screen key resistance targets like AR-V7 [61], thereby optimizing antigen design to reduce screening costs. Regarding the lengthy preparation cycle for personalized mRNA vaccines, Al-mansoori [39] suggested that modular LNPs could enable rapid encapsulation. The successful implementation of these mRNA therapeutic strategies relies on innovations in delivery systems. Specifically, Tiroille et al. [62] developed Nanoblades, virus-like particles that achieved highly efficient editing with zero off-target effects in prostate organoids. Meanwhile, Akbaba et al. [63] designed a Lipo/Cas9-sgRNA system, demonstrating the advantages of cationic liposomes in protecting nucleic acids and enabling efficient delivery.

Regarding novel editors, studies have found that the CRISPR/Cas13 system [64] can directly target RNA for precise editing, providing a new strategy for targeting “undruggable” targets. Cui et al. used aptamer-modified SCORT-Cas13d nanoparticles to target the “undruggable” oncogenic transcription factor homeobox protein B13 (HoxB13) [65], significantly reducing HoxB13 mRNA levels and inhibiting metastatic prostate cancer progression in vivo. Huang et al. designed a TT3-LNP delivered CasRx system capable of targeted degradation of E2F transcription factor 8 (E2F8) mRNA [66], inhibiting the proliferation of AR-negative PCa. Chen Y. et al. Proposed combining the traditional Chinese medicine compound Qingdai Tang (QDT) with the CRISPR-Cas13 system to knock down nitric oxide synthase 3/transforming growth factor beta 1/nuclear receptor coactivator 2 (NOS3/TGFB1/NCOA2) mRNA expression, regulating the phosphoinositide 3-kinase - protein kinase B (PI3K-AKT) [67] pathway at the RNA level, which reversed the inhibitory effect on prostate cancer cells. Lee et al. [68] constructed an aptamer-LNP delivery system targeting programmed cell death ligand 1 (PD-L1), which restored phosphatase and tensin homolog (PTEN) expression to inhibit the PI3K/AKT pathway, thereby significantly inducing apoptosis in CRPC cells and suppressing tumor growth both in vitro and in vivo.

In summary, by optimizing mRNA therapies and innovating delivery systems, CRISPR technology not only enhances the efficacy of existing strategies but also opens up RNA-targeting therapeutic approaches, thereby ushering in a new era for prostate cancer treatment.

## 3 CRISPR in miRNA functional analysis and precision medicine

Within the regulatory network of prostate cancer, there exists a core regulatory framework dominated by mRNA-mediated mechanisms. In addition, there are more subtle and finely tuned regulatory mechanisms governed by microRNAs (miRNAs). Owing to their precise regulatory nature, the latter have emerged as another exemplar for the application of CRISPR technology in ultrasensitive diagnostics and precision therapeutics.

### 3.1 CRISPR defines core miRNA regulatory mechanisms

MiRNAs are a class of non-coding RNAs approximately 18-24 nt in length that primarily fine-tune gene expression at the post-transcriptional level by binding to the 3′-UTR of target mRNAs, mediating their degradation or translational repression, and they exhibit dynamic expression patterns in PCa [7,69]. Using CRISPRa and CRISPR/Cas9 knockout technologies, researchers have directly validated the tumor-suppressive or oncogenic functions of miRNAs [70,71]. Ramalho et al. [72], by knocking out the miR-152-3p target gene transmembrane protein 97 (TMEM97) using CRISPR/Cas9 technology, reciprocally verified that this miRNA exerts a tumor-suppressive role in prostate cancer by inhibiting the mitogen-activated protein kinase/extracellular signal-regulated kinase (MAPK/ERK) pathway and epithelial-mesenchymal transition (EMT). Research by Lovnicki et al. [73], using CRISPR/Cas9 technology, confirmed that LIN28B promotes treatment-induced neuroendocrine prostate cancer (t-NEPC) progression by inhibiting the let-7 miRNA/SRY-box transcription factor 2 (SOX2) axis, thereby relieving suppression of high mobility group AT-hook 2 (HMGA2). Profumo et al. [74] discovered that miR-205, in synergy with a lncRNA, maintains the basal cell phenotype and suppresses prostate cancer progression. Zhang et al. [75], utilizing CRISPRi to suppress miR-3622b-3p, confirmed that it inhibits EMT by targeting apoptosis inducing factor mitochondria associated 2 (AIFM2), suggesting this axis could serve as a therapeutic target for prostate cancer.

Regarding oncogenic miRNAs, Camargo et al. [76], by editing the miR-21 locus with CRISPR/Cas9 technology, confirmed that it enhances the invasiveness of PCa cells and inhibits apoptosis by targeting reversion-inducing cysteine-rich protein with Kazal motifs (RECK), programmed cell death 4 (PDCD4), and matrix metallopeptidase 9 (MMP9). Large-fragment CRISPR editing of specific genomic regions containing miRNA clusters [77] has revealed their synergistic oncogenic roles. Chambers et al. [78] knocked out the entire miR-888 cluster located on the X chromosome using CRISPR, which significantly inhibited PCa cell proliferation and tumor burden. Wang et al. [79], using CRISPR editing, confirmed that miR-1205 promotes castration resistance in prostate cancer by targeting egl-9 family hypoxia-inducible factor 3 (EGLN3).

Merk et al. [80] utilized a novel CRISPR-Cas9 knockout library to identify 49 potentially essential miRNAs that impact cancer cell survival across various tumor cell lines, including the PCa cell line DU145. Furthermore, Chow et al. developed another CRISPR knockout library, miRKOv2, and screened the prostate cancer cell lines DU145 and LNCaP. They also found miR-483 had the most significant impact on PCa cell viability. Further investigation revealed that miR-483-3p [81] maintains prostate cancer cell survival by directly regulating the BCL2 associated transcription factor 1/p53 upregulated modulator of apoptosis/BCL2 antagonist/killer 1 (BCLAF1/PUMA/BAK1) signaling axis to inhibit apoptosis, suggesting miR-483-3p is a potential tumor-specific therapeutic target. Additionally, Jiang et al. [82] performed the first functional mapping of miRNAs in prostate cancer via CRISPR screening, providing a new perspective for systematically studying miRNA regulatory roles in prostate cancer.

### 3.2 CRISPR unravels complex miRNA circuits and drug resistance

CRISPR technology holds unique advantages in dissecting the complex regulatory circuits involving miRNAs, enabling the precise elucidation of their core roles in PCa. Qi et al. [83] activated endogenous cyclin-dependent kinase 13 (CDK13) using CRISPRa and discovered that it forms a self-reinforcing circuit through the circCDK13-miR-212-5p/miR-449a-E2F5 axis, thereby driving PCa progression. In terms of therapy resistance, CRISPR knockout technology has clarified the regulatory roles of miRNAs in key pathways. Yin et al. [84] demonstrated that CRISPR knockout of ataxia telangiectasia mutated (ATM) can reverse enzalutamide resistance mediated by the N-Myc-miR-421 axis. Beyond participating in complex feedback loops, studies have found that miRNAs can bind to the 3′-UTR regions of mRNAs to degrade or inhibit the translation of target genes [85], thereby regulating autophagy and PCa progression (Table 1), and can serve as diagnostic biomarkers for PCa.

**Table 1:** Mechanisms of Key miRNAs in Regulating Autophagy in Prostate Cancer.

miRNA	Target Gene(s)	Regulation of Autophagy	Effect on Prostate Cancer
miR-26b	ULK2^1^	Inhibition	Inhibits proliferation
miR-146b	PTEN →PI3K/AKT/mTOR^2^↑	Inhibition	Promotes progression
miR-205	TP53INP1^3^/RAB27A^4^	Inhibition	Enhances radiosensitivity
miR-381	RELN → PI3K/AKT ↓	Induction	Inhibits proliferation and promotes apoptosis

^1^UNC-51 like autophagy activating kinase 2; ^2^Mechanistic target of rapamycin; ^3^Tumor protein p53 inducible nuclear protein 1;^4^RAS-associated protein RAB27A.

### 3.3 CRISPR enables ultrasensitive miRNA diagnostics

In precision medicine, the ability to detect extremely low concentrations of cancer signals in blood during the early stages of disease is crucial for diagnostic efficacy [86,87]. However, the extremely low abundance of these signal molecules makes them difficult to capture using conventional methods. In recent years, proteins from the CRISPR gene editing family, specifically Cas12a and Cas13a [88,89], have been found to possess not only gene-editing capabilities but also function as highly efficient miRNA detection tools. These proteins, upon recognizing specific miRNA targets, become activated and subsequently cleave reporter molecules indiscriminately, bringing revolutionary breakthroughs to ultrasensitive diagnostics.

To address the challenge of detecting extremely low miRNA levels in blood, research has combined the specific recognition capability of CRISPR/Cas12a/Cas13a technology with various nucleic acid amplification techniques for signal enhancement, ultimately achieving ultrasensitive miRNA detection. For example, Zhu et al. [90] developed the CENTER platform, which integrates CRISPR/Cas12a with strand displacement amplification, achieving a detection limit as low as 34 aM for serum miR-141. Ma et al. [91] innovated the miRoll-Cas system, which amplifies signals via rolling circle transcription, enhancing sensitivity by 1000-fold compared to traditional methods and enabling single-base mismatch discrimination.

Achieving simple and intuitive result readout is key to the clinical translation of these ultrasensitive detection technologies. The development of diversified readout platforms provides solutions for this requirement. Wang et al. [92] combined CRISPR/Cas12a with programmable DNA nanoswitches and a gold nanostar reporting platform, achieving highly sensitive detection of miRNA-375. Jiang et al. developed a platform coupling CRISPR/Cas12a with hyperbranched rolling circle amplification (HRCA), enabling visual readout through gold nanoparticle aggregation-induced color change with a detection limit at the fM level [93], offering potential for point-of-care testing (POCT).

By integrating specific recognition, signal amplification, and diversified readout technologies, the CRISPR platform establishes a unique technological pathway for highly sensitive and specific miRNA diagnostics, demonstrating broad potential for clinical application.

### 3.4 CRISPR facilitates precision miRNA therapeutics

CRISPR-based genome editing technology provides precise tools for directly targeting pathogenic miRNAs in prostate cancer. Zhang et al. [75] constructed an inducible CRISPRi system that reversibly suppresses miR-3622b-3p transcription, effectively blocking the EMT process. Dart et al. [94] permanently knocked out the miR-221 locus using CRISPR/Cas9, significantly inhibiting cancer cell migration capability. Wang et al. [79] successfully knocked out miR-1205 using CRISPR/Cas9 technology, revealing that it inhibits cell proliferation and promotes castration-resistant prostate cancer progression by targeting EGLN3. Furthermore, CRISPR technology can also reveal upstream regulatory mechanisms of miRNAs. Takao et al. using a PTEN-KO model constructed via CRISPR/Cas9, found that PTEN loss leads to upregulated expression of mmu-miR-210-3p [95], collectively promoting EMT, angiogenesis, and immunosuppression.

Breakthroughs in delivery technology are crucial for realizing the therapeutic potential of CRISPR-miRNA interventions. Valiunas et al. [96] used CRISPR/Cas9 technology to confirm that the gap junction protein connexin 43 (Cx43) serves as a core channel for intercellular miRNA delivery. Lipid nanoparticles encapsulating anti-miR-375 can restore protein tyrosine phosphatase non-receptor type 4 (PTPN4) expression and enhance enzalutamide sensitivity [97]. Combined intervention strategies demonstrate the broad prospects of CRISPR-miRNA therapy. Studies have found that miR-205 [97] can inhibit cancer-associated fibroblast-induced EMT and enhance the killing effect of docetaxel on tumor stem cells. Gold nanoparticles delivering miR-34a combined with B7-H3 chimeric antigen receptor T-cell (CAR-T) therapy can synergistically eliminate radiotherapy-resistant cells [97]. These findings demonstrate that CRISPR technology has significantly advanced miRNA research and fostered innovation in therapeutic strategies for PCa.

## 4 CRISPR in circRNA biology and clinical translation

Circular RNAs (circRNAs) are defined as a special class of non-coding RNA molecules characterized by a closed circular structure [[98], [99], [100]]. Due to their structural stability, circRNAs have been established as a new focus in prostate cancer research. This section is focused on elucidating how CRISPR technology is utilized to systematically dissect circRNA functions, reveal their upstream regulatory networks, drive diagnostic innovation, and thereby promote its translation into clinical diagnostics and therapy.

### 4.1 CRISPR decodes circRNA molecular functions

CRISPR/Cas9 technology has been employed to provide key genetic evidence for directly validating molecular functions of circRNAs, unequivocally confirming their classic role as competitive endogenous RNAs (ceRNAs). ceRNAs are a class of RNA molecules that include circRNAs and lncRNAs [101]. They function as molecular sponges by competitively binding to shared miRNAs, thereby indirectly regulating the expression of downstream target genes and forming a complex post-transcriptional regulatory network [102]. Weidle et al. [103] discovered that targeted intervention of circ-zinc finger protein 609 (circZNF609) releases its sequestration of miR-501-3p, thereby promoting HK2-mediated glycolysis and radioresistance. Similarly, circCDK13 was found to relieve E2F5 suppression through sponging miR-212-5p/miR-449a [83], while circ-cyclin B2 (circCCNB2) induces autophagy and radioresistance by acting as a sponge for miR-30b-5p [85]. Furthermore, CRISPR studies have revealed non-canonical functions involving circRNA-protein interactions. For instance, circ0003258 promotes castration resistance by binding insulin-like growth factor 2 mRNA binding protein 3 (IGF2BP3) to stabilize histone deacetylase 4 (HDAC4) mRNA, whereas circ-formin 2 (circFMN2) [103] was identified to enhance metastatic potential through recruitment of human antigen R (huR) to suppress Krüppel-like factor 2 (KLF2) transcription.

### 4.2 CRISPR manipulates circRNA for intervention

Compared to traditional RNAi, the CRISPR system provides a tool with higher specificity for circRNA functional studies. In loss-of-function studies, the CRISPR system enables precise interventions ranging from transcript degradation to circularization inhibition. Teng et al. [104] designed CRISPR/Cas13d to directly degrade circ-rhabdomyosarcoma 2 associated transcript (circRMST), confirming its role as an independent functional entity driving neuroendocrine differentiation. In research by Weidle et al. [103], genomic flanking sequences of circ-midline 1 (circMID1) and circ-lysine demethylase 4 A (circKDM4A) were knocked out using CRISPR/Cas9, effectively suppressing tumor growth and clarifying their ceRNA mechanisms. Gain-of-function studies demonstrate the therapeutic potential of CRISPR technology. Employing the CRISPR/dCas9-VPR system to specifically overexpress circRNA17 [103] successfully suppressed AR-V7 and enhanced enzalutamide sensitivity, providing a new therapeutic strategy.

### 4.3 CRISPR achieves ultrasensitive circRNA detection

CRISPR-based detection technology provides a highly specific tool for the clinical diagnosis of circRNAs. The CrisprZyme technology developed by Broto et al. [105], which utilizes Cas13 to cleave circ-aurora kinase A (circAURKA)-specific back-splice junctions combined with a nanozyme signal amplification system, was successfully applied to achieve high-sensitivity discrimination between neuroendocrine prostate cancer and prostatic adenocarcinoma.

### 4.4 CRISPR screens identify upstream circRNA regulators

Genome-wide CRISPR screening technology has systematically revealed the core regulatory network of circRNA biogenesis in prostate cancer. Using the GeCKO v2 library for CRISPR screening, Fei et al. identified the RNA-binding protein HNRNPL as a key regulatory gene in prostate cancer, which directly regulates the formation of 232 circRNAs [29], including AR-related circRNAs, through binding to CA-repeat sequences. This finding was further validated by Wang et al. [106], whose research demonstrated that small molecule inhibitors targeting CA-repeat sequences can effectively block HNRNPL function and inhibit circRNA production, with this strategy having advanced to the preclinical development stage. These studies collectively form a complete translational pathway from “CRISPR screening technology identifying core factors” to “developing targeted therapeutic strategies".

## 5 CRISPR in lncRNA 3D regulation and clinical translation

Long non-coding RNAs (lncRNAs) are a class of RNA molecules exceeding 200 nucleotides in length that generally do not encode proteins, playing central roles in gene regulation at multiple levels including transcriptional and post-transcriptional processes [107,108]. Studies have demonstrated that lncRNAs serve important regulatory functions in prostate cancer [109]. This section focuses on how CRISPR technology deciphers lncRNA functions, reveals their genetic regulatory basis, and promotes their clinical translation.

### 5.1 CRISPR elucidates lncRNA functional mechanisms

CRISPR technology provides multiple precise strategies for directly analyzing the functional mechanisms of lncRNAs. For ceRNA functional validation, Xiao et al. [110] employed CRISPR genome editing to knockout the Y chromosome lncRNA testis-specific transcript Y 15 (TTTY15), confirming that it drives tumor progression by sponging let-7 family miRNAs, thereby releasing the inhibition of cyclin-dependent kinase 6/fibronectin 1 (CDK6/FN1). Vitiello et al. [111] adopted a dual CRISPR strategy to intervene with PTENP1 at both genomic and RNA levels, providing the most reliable genetic evidence for its role as a ceRNA for PTEN. For lncRNAs with complex functions, CRISPR technology can also reveal non-ceRNA mechanisms. Through targeted knockout of MALAT1 using CRISPR/Cas9, Ahmadi et al. [112,113] discovered that it drives tumor proliferation by regulating cell cycle and apoptosis genes, independent of ceRNA mechanisms.

### 5.2 CRISPR reveals the genetic basis of lncRNA regulation

Research indicates that prostate cancer pathogenesis is associated with genomic SNPs, with substantial evidence suggesting that SNPs exert their effects through the regulation of lncRNAs, though the specific mechanisms remain incompletely understood. CRISPR base editing technology has provided direct evidence for establishing the causal relationship between SNPs and lncRNA expression regulation. Expression quantitative trait loci (eQTLs) represent genetic loci [114,115] that influence gene expression levels. Studies have revealed that cis-eQTLs may directly affect transcription [116] by regulating sequences such as promoters and enhancers. Through CRISPR-mediated editing of the rs11672691 locus, Gao et al. [117] confirmed that the G allele upregulates the oncogenic lncRNA prostate cancer associated transcript 19 (PCAT19) by enhancing homeobox protein A2 (HOXA2) binding through long-range interactions. Trans-eQTLs [118] refer to genetic variations located in distal genomic regions that regulate target gene expression. Regarding trans-eQTL mechanisms, Bicak et al. [119] employed CRISPR/Cas9 to knockout the risk SNP rs10993994, systematically delineating its role in mediating the tumor-specific expression of lncRNA small nucleolar RNA host gene 11 (SNHG11) through the regulation of MSMB and nuclear receptor coactivator 4 (NCOA4) within a complex network.

### 5.3 CRISPR screens systematically identify functional lncRNAs

CRISPR-based screening studies have further revealed the regulatory network of lncRNAs within the three-dimensional genomic context. By integrating genome-wide association study (GWAS) with CRISPR screening systems, Luo et al. [120] discovered that the 7p15.2 risk region promotes PCa invasion by regulating the lncRNA HOXA distal transcript (HOTTIP) through an inhibitory chromatin loop, subsequently activating the Hippo pathway. A large-scale CRISPR screening conducted by Guo et al. [121] further revealed that approximately 50% of PCa risk loci are located within lncRNA regulatory regions, such as rs7463708-T which drives advanced disease progression through activation of prostate cancer associated transcript 1 (PCAT1). Through the CADTAD pipeline integrating Hi-C data with CRISPR validation, Rao et al. [122] screened 241 functional lncRNAs from prostate cancer-specific topologically associating domains, including 149 oncogenic and 85 tumor-suppressive types, establishing a foundation for the precise targeting of lncRNAs.

### 5.4 CRISPR advances lncRNA clinical translation

Functional studies using CRISPR have accelerated the clinical translation of lncRNAs. In the field of diagnosis and detection, prostate cancer antigen 3(PCA3) [121] has been approved by the FDA as a urinary detection biomarker, while MALAT1 and TTTY15 [29,104] are emerging as promising novel diagnostic biomarkers. For prognosis assessment, models such as the MALAT1/HOXB-AS3 combination and the rs11672691 GG genotype with high PCAT19 expression [29,111] have been demonstrated to effectively predict patient outcomes. Regarding therapeutic strategies, CRISPRi and CRISPR/Cas9 genome editing technologies, when combined with delivery systems such as LNPs, offer diverse and precise approaches for targeting oncogenic lncRNAs [112,113,123]. Notably, CRISPR technology has also been employed to elucidate the interactions between lncRNAs and other critical pathways. Through CRISPR technology, Park et al. [124] confirmed that inhibition of the dopamine receptor dopamine receptor D2 (DRD2) suppresses the formation of prostate cancer stem cells via the AMP-activated protein kinase (AMPK) pathway, providing new targets for combination therapies.

## 6 CRISPR in eRNA regulatory networks and therapy

Enhancer RNAs (eRNAs) represent a significant category of non-coding RNAs [125]. These short RNA transcripts are produced from genomic enhancer regions and contribute to prostate cancer pathogenesis through target gene regulation. CRISPR gene editing has been established as a highly effective tool [126] for deciphering non-coding RNA functions via precise eRNA targeting. This section elaborates on how CRISPR technology systematically investigates eRNA functions, reveals their upstream regulatory networks, and facilitates clinical translation.

### 6.1 CRISPR characterizes eRNA features and regulation

CRISPR technology serves as a key tool for directly dissecting the bidirectional transcription characteristics and functional patterns of eRNAs. Studies have demonstrated that eRNAs comprise sense and antisense strands produced through bidirectional transcription from enhancer regions [[119], [120], [121], [122]], with their transcriptional activity closely associated with chromatin accessibility and precise localization within topologically associating domains (TADs) through high-resolution chromatin interaction mapping. Functionally, eRNAs operate through cis-regulation, as exemplified by LTFe which directly regulates its host gene lactoferrin (LTF) via spatial proximity, establishing the critical LTFe-LTF regulatory axis [127]. Antisense eRNAs can employ allele-specific mechanisms to recruit DNA methyltransferase 1 (DNMT1) to gene termini in an AR-dependent manner, suppressing neighboring gene expression through localized DNA methylation [128]. Simultaneously, eRNAs also execute trans-regulatory functions, illustrated by prostate-specific antigen (PSA) eRNA which activates the positive transcription elongation factor b (P-TEFb) complex through binding to CYCLIN T1 protein, subsequently phosphorylating RNA polymerase II to promote global gene transcription, a process independent of its genomic location [129].

### 6.2 CRISPR maps eRNA-Mediated chromatin interactions

The advancement of CRISPR technology systems has provided powerful methodological support for delineating the three-dimensional chromatin conformation mediated by eRNAs. Research has revealed that eRNAs serve as critical drivers of chromatin loop formation. For instance, HPSE eRNA directly facilitates chromatin loop formation by binding to the heterogeneous nuclear ribonucleoprotein U (hnRNPU)/p300/early growth response protein 1 (EGR1) complex, thereby activating the HPSE gene promoter [130]. Chromatin interaction studies have further uncovered a unique “dual-loop architecture”. In this structure, sense, eRNAs promote enhancer-promoter interactions. Antisense eRNAs connect enhancers with gene terminal regions. This architecture has been demonstrated to be essential for neighboring mRNA expression [128]. Through integration of CRISPR with high-resolution Hi-C technology, it has been confirmed that eRNA-associated enhancer-promoter interactions are frequently enriched at CTCF binding sites, nucleosome-depleted regions, and active histone mark regions, with these interactions undergoing significant reprogramming in cancer cells [131,132].

### 6.3 CRISPR drives eRNA clinical translation

Functional studies based on CRISPR are accelerating the translation of eRNAs from fundamental discoveries to clinical applications. In the diagnostic field, large-scale omics studies have identified numerous eRNA quantitative trait loci (eRNA-QTLs) significantly associated with cancer risk and patient survival, providing valuable resources for biomarker development [133,134]. In the therapeutic area, functional studies have confirmed that depletion of key eRNAs such as LTFe, cyclin D1 eRNA (CCND1e), and small nuclear RNA activating complex polypeptide 1 eRNA (SNAPC1e). They have been achieved through CRISPRi or shRNA knockdown, leads to significant downregulation of their target oncogenes and effectively suppresses prostate cancer cell proliferation [133,135]. Furthermore, the direct targeting of pathogenic eRNAs or their regulated oncogenic transcripts using RNA-targeting technologies like CRISPR-CasRx has demonstrated substantial anti-tumor effects in preclinical models, establishing a solid foundation for developing novel therapies targeting eRNA networks [[129], [130], [131]].

## 7 CRISPR-mediated m^6^A editing, regulation, and therapy

N^6^-methyladenosine (m^6^A), the most abundant mRNA modification, plays a central role in gene expression regulation by dynamically and reversibly controlling RNA stability, splicing, translation, and degradation [8,136]. Studies demonstrate that m^6^A modification exerts dual roles in prostate cancer by regulating the stability and functions of both mRNAs and non-coding RNAs. The YTH N^6^-methyladenosine RNA binding protein 1 (YTHDF1) axis is promoted to enhance the translation of oncogenes like polo-like kinase 1 (PLK1), thereby driving tumor progression [137]. Concurrently, evidence confirms that YTH N^6^-methyladenosine RNA binding protein 2 (YTHDF2) mediates the degradation of tumor suppressor genes phospholysine phosphohistidine inorganic pyrophosphate phosphatase (LHPP) and NK3 homeobox protein 1 (NKX3-1), leading to AKT phosphorylation activation and promoting tumor progression [138]. Methyltransferase-like protein 3 (METTL3), heterogeneous nuclear ribonucleoprotein A2/B1 (HNRNPA2B1), and other factors further participate in PCa pathogenesis by modifying miRNAs, lncRNAs, and circRNAs, forming intricate ceRNA networks.

Therapeutic strategies targeting m^6^A are emerging as a new direction for precision therapy in PCa. Small-molecule inhibitors such as STM2457 can target METTL3, inhibiting PCa cell proliferation and migration, and synergize with the PARP inhibitor Olaparib to enhance anti-tumor effects [139]. Among these approaches, CRISPR-Cas13 technology demonstrates unique advantages. By constructing dCas13b-fat mass and obesity-associated protein (FTO) or dCas13b- AlkB homolog 5 (ALKBH5) fusion proteins, selective removal of m^6^A methylation at specific RNA sites is achieved, thereby regulating the stability and translation efficiency of target mRNAs [140]. Furthermore, Zhao et al. developed the optogenetic system PAMECR, which combines CRISPR-Cas13 with light-induced dimerization to enable spatiotemporally controlled m^6^A editing, providing an efficient tool for “epitranscriptome engineering” [141]. Additionally, Cas13 can be utilized for detecting m^6^A-modified RNAs in liquid biopsies, aiding in early diagnosis and dynamic monitoring of PCa [[142], [143], [144]].

## 8 Conclusion and perspectives

**Fig. 4:**
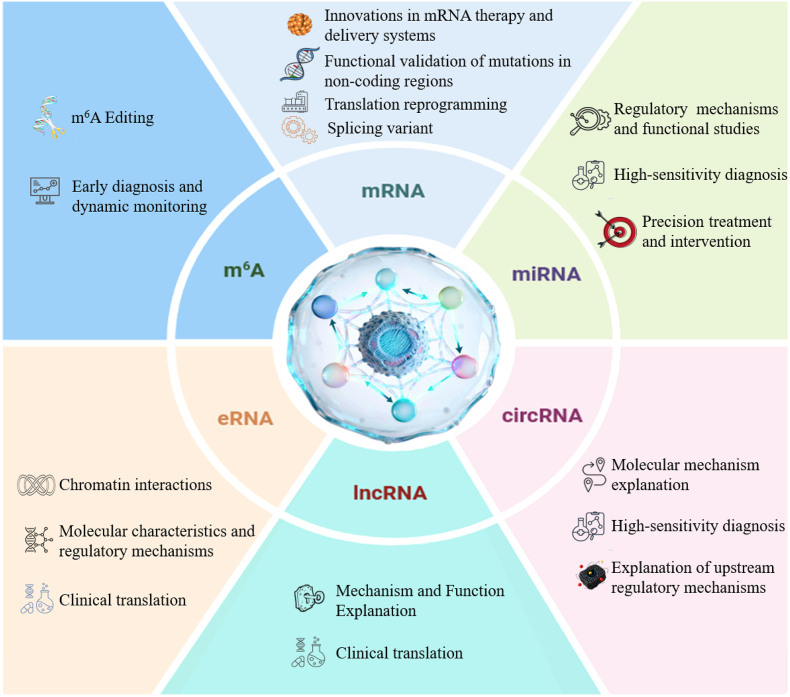
**Mechanistic Research and Clinical Translation of CRISPR Technology on mRNA, miRNA, circRNA, lncRNA, eRNA, and m6A Modification in Prostate Cancer.** The mechanistic research and clinical translation of CRISPR technology for various RNA molecules mRNA, miRNA, circRNA, lncRNA, eRNA, and m^6^A Modification in prostate cancer. It highlights the versatility of CRISPR/Cas systems by illustrating diverse strategies for knocking out, modifying, or activating specific RNAs. The figure then directly links these upstream fundamental discoveries to downstream clinical applications. Translational directions include targeted therapy of oncogenic mRNAs, diagnostic biomarker development through miRNA editing, functional regulation of circRNAs and lncRNAs for liquid biopsy applications, interference with eRNA enhancer activity for epigenetic therapies, and rewriting of m^6^A marks to control mRNA stability and translation. The diagram shows a direct pathway from basic research to clinical diagnostics and therapeutic development.

Prostate cancer progression is driven by a complex and multi-layered RNA regulatory network. This review summarizes how CRISPR technology systematically decodes this network. It provides critical functional insights and identifies new therapeutic targets. CRISPR tools have precisely defined the roles of various RNA molecules. For mRNA, CRISPR screens revealed key splicing variants such as AR-V7 that drive castration resistance. Cas13 systems enable direct RNA targeting and offer new strategies for degrading oncogenic transcripts. For non-coding RNAs, high-throughput screens defined core oncogenic and tumor-suppressive miRNAs. CRISPR-based detection platforms achieved ultrasensitive liquid biopsy for miRNAs and circRNAs. Knockout and activation studies clarified how circRNAs and lncRNAs function as molecular sponges and regulate transcription and chromatin architecture. For eRNAs and m^6^A modifications, CRISPR tools mapped intricate chromatin interactions and enabled site-specific manipulation of RNA methylation to control transcript stability. These mechanistic insights propel clinical translation. CRISPR-validated neoantigens optimize mRNA vaccine design. Advanced delivery systems improve targeting efficiency. The integration of CRISPR diagnostics with multi-omics data promises earlier detection and better patient stratification (Fig. 4).

Several key directions will shape future progress in this field. Novel CRISPR systems must achieve higher editing efficiency and better safety profiles [145]. Organ-selective delivery platforms are essential for precise in vivo therapy [22,146]. CRISPRa/i tools can reconstitute pathogenic splicing networks and overcome drug resistance through combination regimens. CRISPR-based neoantigen identification will accelerate mRNA vaccine development. Ultrasensitive CRISPR detection platforms are poised to become routine liquid biopsy tools. Base editing, CRISPRi, and domain-specific targeting further expand the therapeutic toolbox for non-coding RNAs [58,90,147,148]. Meanwhile, integrating multi-omics data to build prostate cancer-specific regulatory networks and exploring synergy between CRISPR and existing therapies will pave the way toward personalized precision medicine [[149], [150], [151]].

However, numerous challenges remain in the field. Off-target risks, uneven editing efficiency, insufficient delivery specificity, and in vivo immunogenicity have not been fully resolved [[152], [153], [154]]. The dynamic nature of RNA networks and their interactions with epigenetics and 3D genomics are still poorly understood. Effective tools for integrated multi-omics analysis are also lacking [155]. For clinical translation, the high cost and lengthy timelines of personalized strategies require attention. Long-term safety and ethical concerns also demand thorough evaluation [156]. This review has focused on core applications of CRISPR in prostate cancer RNA research. Subsequent studies should expand to emerging non-coding RNA families such as piRNAs and snoRNAs. This will help construct a more comprehensive RNA regulatory atlas for prostate cancer [[157], [158], [159]]. Overcoming these hurdles will require continued technological iteration, multi-omics integration, and robust preclinical validation. Only then can CRISPR-RNA therapies deliver more personalized, safe, and effective strategies for prostate cancer patients.

## CRediT authorship contribution statement

**Song Zhu:** Writing – review & editing, Writing – original draft, Visualization, Investigation, Data curation, Conceptualization. **Gao Ni:** Writing – review & editing, Writing – original draft, Visualization, Investigation. **Pinjie Zhang:** Writing – original draft, Validation, Data curation, Writing – review & editing, Writing – original draft, Visualization, Supervision, Data curation, Conceptualization. **Yili Yang:** Writing – original draft. **Hongxiang He:** Writing – original draft. **Junfeng Jiang:** Writing – review & editing, Writing – original draft, Visualization, Supervision, Funding acquisition, Data curation, Conceptualization. **Zhang Li:** Writing – review & editing, Writing – original draft, Visualization, Supervision, Data curation, Conceptualization.

## Funding

This research was funded by the Chinese Natural Science Foundation (82472732), “Basic + X″ Collaborative Research Program Foundation of School of Basic Medical Sciences, NMU (JCHZJL-007), 10.13039/100017950Shanghai Municipal Health Commission - Medical New Technology Research and Transformation Seed Program (2025ZZ2019), Research Project of Naval Medical University (2023MS0001).

## Declaration of competing interest

The authors declare that they have no known competing financial interests or personal relationships that could have appeared to influence the work reported in this paper.
